# Circulating Exosome Involves in the Pathogenesis of Autoimmune Thyroid Diseases Through Immunomodulatory Proteins

**DOI:** 10.3389/fimmu.2021.730089

**Published:** 2021-11-11

**Authors:** Xi Jia, Tianyu Zhai, Jin-an Zhang

**Affiliations:** ^1^ Department of Endocrinology, Shanghai University of Medicine & Health Sciences Affiliated Zhoupu Hospital, Shanghai, China; ^2^ Department of Endocrinology, Zhongshan Hospital, Fudan University, Shanghai, China

**Keywords:** autoimmune thyroid diseases (AITDs), exosome, proteomics, Graves’ disease, Hashimoto’s thyroiditis (HT)

## Abstract

Autoimmune thyroid diseases (AITDs) are chronic organ-specific autoimmune diseases, mainly including Graves’ disease (GD) and Hashimoto’s thyroiditis (HT). Exosomes, as extracellular vesicles, contain a variety of biologically active substances that play a role in information exchange, thereby affecting the occurrence and progression of diseases. However, it is unclear whether exosomes are involved in the pathogenesis of AITDs. In this study, the role of exosomes in AITDs was explored from a proteomics perspective. Plasma exosomes were isolated from 12 patients with GD, 10 patients with HT, and seven normal controls (NC). Protein profiles were detected using the data-independent acquisition (DIA) method and analyzed to investigate changes in plasma exosome proteins. In the setting of GD, 11 proteins were upregulated while 197 proteins were downregulated compared with healthy people. Among them, MAP1S (log_2_ FC = 4.669, *p* = 0.009) and VAMP8 (log_2_ FC = 3.216, *p* = 0.003) were the most significantly upregulated, and RSU1 (log_2_ FC = −6.797, *p* = 0.001), ACTB (log_2_ FC = −4.795, *p* < 0.001), and CXCL7 (log_2_ FC = −4.674, *p* < 0.001) were the most significantly downregulated. In the cases of HT, HGFL (log_2_ FC = 2.766, *p* = 0.001), FAK1 (log_2_ FC = 2.213, *p* < 0.001), and PTN12 (log_2_ FC = 1.624, *p* < 0.001) were significantly upregulated, while PSMF1 (log_2_ FC = −3.591, *p* < 0.001), PXL2B (log_2_ FC = −2.622, *p* = 0.001), and CYTM (log_2_ FC = −1.609, *p* < 0.001) were the most downregulated. These differential proteins were mainly enriched in the immune system and metabolic system, indicating that plasma exosomes may play an important role in systemic immune imbalance in AITDs.

## Introduction

Autoimmune thyroid diseases (AITDs) are the most common chronic organ-specific autoimmune diseases in the world, mainly including Graves’ disease (GD) and Hashimoto’s thyroiditis (HT) (1, 2). The etiology of AITDs is still not fully discovered and involves multiple factors such as environment, gender, genetics, and immunity (3, 4). Exosomes are membrane-bound extracellular vesicles with diameter in the range of 50–150 nm, which are released into body fluids by various cells under both normal and pathophysiological conditions (5). Although they were initially considered as “garbage bags” to clear non-functional molecules of cells (6), more and more evidence has shown that they actually serve as carriers of various biologically active substances such as mRNA, miRNA, proteins, and lipids, mediating materials and signal exchanges between cells (7, 8). These active substances in exosomes are the key to make exosomes participate in various pathophysiological processes, such as embryo development, stem cell differentiation, tumor metastasis, drug resistance, and immune system activation (9–11). Many studies have pointed out that in autoimmune diseases, such as inflammatory bowel disease (IBD) (12, 13), rheumatoid arthritis (RA) (14), primary Sjogren’s syndrome (Pss) (15), and multiple sclerosis (MS) (16), the protein profiles of exosomes have significantly changed with the disease progression. The concepts of “autoimmune tautology” and “autoimmune mosaic” state that there are often shared characteristics between autoimmune diseases. However, the role of exosomes in the occurrence and natural history of AITDs development is still unknown.

Data-independent acquisition (DIA) is an emerging advanced protein profiling detection and analysis technique in proteomics, as it does not need the selection of precursor ions from mass spectrometry (MS) spectra for peptide fragmentation but instead enables fragmentation in all ionized peptide samples (17). This method obviously benefits from a significantly increased MS/MS signal, which is beneficial for both the identification and quantification of protein analysis (18). The purpose of this study was to use DIA technique to investigate changes in circulating exosomal protein profiles in patients with AITDs to explore the role of exosomes in the etiology of AITDs.

## Material and Methods

### Study Design

The differential expression profile of exosomal proteins in plasma of patients with AITDs was investigated in a case–control study. Patients were consecutively enrolled from the Department of Endocrinology at Zhoupu Hospital; and gender- and age-matched healthy normal controls (NC) were recruited from the Health Check-up Center of the same hospital. To exclude the effects of other interference factors as much as possible, subjects with infectious diseases within 3 months and any other autoimmune and chronic diseases were excluded. The clinical characteristics of all subjects including disease duration, family history, the degree of Grave’s ophthalmopathy, and goiter were collected, as well as the serological data including serum levels of thyroid-stimulating hormone (TSH) receptor antibody (TRAb) (IU/L), thyroid peroxidase antibody (TPOAb) (IU/ml), thyroglobulin antibody (TGAb) (IU/ml), free triiodothyronine (FT3) (pmol/L), free thyroxine (FT4) (pmol/L), and TSH (μIU/ml).

### Diagnostic Criteria and Subgroup Settings

Patients who presented with thyrotoxicosis and were TRAb-positive were diagnosed as GD. Among them, patients who were initially confirmed to have GD and treatment-naive, which reflects the most realistic immune status in the GD state, were assigned to the newly diagnosed GD (NG) subgroup, while patients who had been diagnosed with GD and continued to receive standard antithyroidal therapy at least for 3 years, but still had TRAb greater than 1.75 IU/L, were assigned to the refractory GD (RG) subgroup. Patients who were TGAb positive or TPOAb positive and had high TSH level with clinically obvious hypothyroidism symptoms were diagnosed as HT.

### Exosome Extraction and Identification

A total of 5 ml of peripheral venous blood from each subject was collected in EDTA anticoagulation tubes. After centrifugation at 2,000 rpm for 5 min at 4°C, 1 ml of the upper clear plasma was aspirated and stored −80°C for future use. To isolate exosomes, all plasma samples were simultaneously thawed to 4°C and centrifuged at 4°C for 10 min at 2,000 *g*, 20 min at 8,000 *g*, and 1 h at 20,000 *g*. At the end of each centrifugation, the supernatants were collected for the next centrifugation. After the final centrifugation, the supernatants were subjected to filtration with 0.22-μm filters to remove any visible precipitates and lipids. The filtrates were then diluted fourfold with phosphate-buffered saline (PBS) in tubes and subjected to ultracentrifugation at 1,500,000 *g* at 4°C for 2 h after making sure that the weight difference of each tube was less than 0.01 g. The pelleted exosomes were resuspended in 1 ml of PBS and subjected to ultracentrifugation again at the same condition.

### Data-Dependent Acquisition Spectral Library, Protein Quantification, and Data-Independent Acquisition Proteomics Technique

The extracted exosomes were lysed with 100 μl of sodium dodecyl sulfate (SDS)-free L3 lysis buffer supplemented with 1× enzyme inhibitor cocktail by pipetting up and down for at least 50 times. After being regulated to a final concentration of 10 mM, samples were incubated in a water bath at 37°C for 45 min. After being cooled to room temperature, samples were treated with 20 mM of iodoacetamide in the dark for 30 min to obtain exosome proteins. Protein concentrations were measured using the Bradford method, and their integrity was examined using polyacrylamide gel electrophoresis.

Protein profile was analyzed using the next-generation label-free quantitative proteomics technology under the DIA (also known as SWATH) mode, which is considered as an ideal differential proteomics analysis and enables accurate and highly repeatable quantification for large amounts of proteins per sample. The DIA analysis pipeline contains three essential steps, namely, spectral library construction, large-sample data acquisition, and data analysis. The spectral library was constructed from samples of interest using data-dependent acquisition (DDA) technique. MaxQuant was then used to carry out database search identification process and obtain all detectable non-redundant high-quality MS/MS spectral information as DIA spectral library. To improve protein identification and quantification and effectively avoid convolution, data analysis was conducted using Spectronaut™, in which iRT peptides were used for retention time calibration and the mProphet scoring algorithm was integrated to accurately reflect the matching level of ion pairs. Then, false-positive control was performed based on the target-decoy model with false discovery rate (FDR) of 1% applicable to SWATH-MS to obtain significant quantitative results. Data quality was evaluated based on intra-group coefficient of variation (CV), principal component analysis (PCA), and quantitative correlation of samples. Quality control samples were inserted intermittently between the continuous original samples to ensure stability and repeatability of the experiment.

### Statistical Analysis

Statistical analysis was performed using R software (version 4.0.4) and STATA (12.0, StataCorp, USA). Continuous variables were calculated as mean ± SD. Differences between groups were analyzed using independent samples t-test, and non-normal distribution data were analyzed using the Mann–Whitney U tests. Differences with both *p*-value and *Q* value (adjusted for the FDR) <0.05 were considered as statistically significant. Significant differences in proteins or peptides were statistically evaluated using MSstats, a widely used R package from the Bioconductor repository in DIA quantitative experiments with linear mixed-effects model as the core algorithm. The possible functions of the differential proteins between different groups were analyzed using Gene Ontology (GO) enrichment analysis, Kyoto Encyclopedia of Genes and Genomes (KEGG) pathway enrichment analysis, and EuKaryotic Orthologous Groups (KOG) analysis.

## Results

### Demographic Information and Clinical Features

A total of seven patients with newly diagnosed GD, five patients with refractory GD, 10 patients with HT, and seven healthy controls were enrolled in the study. The clinical and serological data of those subjects are summarized in **Table 1**. The mean age of the total patients with GD, HT, and healthy controls were 41.4, 35.8, and 37.4 years, respectively. The treatment duration in refractory GD patients was 5.2 ± 1.6 (mean ± SD) years with an average TRAb level of 15.3 IU/L, while the patients with newly diagnosed treatment-naive GD had slightly higher TRAb with an average level of 17.7 ± 6.9 IU/L. All patients who have HT had elevated TSH, TPOAb, and TGAb levels.

**Table 1:** Clinical characteristics of all subjects.

	HT	GD			NC
		Total	NG	RG	
Sample size (n)	10	12	7	5	7
Sample ID	1–10	11–22	11–17	18–22	23–29
Gender (n; male/female)	5/5	5/7	3/4	2/3	3/4
Age (year)	35.8 ± 9.2	41.4 ± 6.3	37.4 ± 3.7	45.4 ± 5.53	38.4 ± 9.2
Duration (year)	4.2 ± 2.1	2.2 ± 2.8	0	5.2 ± 1.6	N/A
Family history (n; male/female)	3/3	3/5	1/3	2/2	N/A
GO (n; male/female)	0/0	2/2	1/1	1/1	N/A
Goiter degree (n; male/female)					
≤I	4/5	2/2	1/1	1/1	3/4
≥II	0/1	3/5	2/3	1/2	0/0
TRAb (IU/L)	2.4 ± 0.9	16.7 ± 6.6	17.7 ± 6.9	15.3 ± 5.0	11.32 ± 7.5
TPOAb (IU/ml)	899.6 ± 210.3	491.5 ± 368.9	414.7 ± 334.1	599.1 ± 358.1	228.3 ± 263.4
TGAb (IU/ml)	538.3 ± 245.2	688.1 ± 1,028.8	452.2 ± 220.9	1,018.4 ± 1,508.3	843.7 ± 1,182
FT3 (pmol/L)	3.7 ± 2.8	18.8 ± 13.8	22.2 ± 11.9	14.1 ± 15.0	16.67 ± 14.9
FT4 (pmol/L)	14.8 ± 5.6	39.0 ± 23.3	41.3 ± 12.0	35.81 ± 32.55	39.4 ± 30.3
TSH (µIU/ml)	3.5 ± 1.3	0 ± 0.01	0 ± 0.01	0.00 ± 0.00	0.2 ± 0.2

The continuous variables are shown in mean ± SD.

HT, Hashimoto’s thyroiditis; NG, newly diagnosed Grave’s disease; RG, refractory Grave’s disease; NC, normal control; GO, Grave’s ophthalmopathy; TSH, thyroid-stimulating hormone; TRAb, TSH receptor antibody; TPOAb, thyroid peroxidase antibody; TGAb, thyroglobulin antibody; FT3, free triiodothyronine; FT4, free thyroxine.

### Exosome Identification, Protein Consistency Detection, and Quality Control

The MS data of 29 samples were acquired, and quantitative statistics of each sample using Spectronaut™ are shown in **Supplementary Table 1**. Qualification of these data based on CV, PCA and quantitative correlation of samples indicates that these data are in good quality, stability, and repeatability (**Supplementary Figure 1**).

### Differential Proteins Between Autoimmune Thyroid Diseases and Healthy Controls

A total of 8,251 peptides and 1,319 proteins were detected. Among these proteins, 733 proteins were unchanged, 11 proteins were downregulated, and 197 proteins were upregulated in the total GD group compared with the NC group, with MAP1S (log_2_ FC = 4.669, *p* = 0.009, *Q* = 0.004) and VAMP8 (log_2_ FC = 3.216, *p* = 0.003) being the most significantly upregulated, and RSU1 (log_2_ FC = −6.797, *p* = 0.001, *Q* = 0.010), ACTB (log_2_ FC = −4.795, *p* < 0.001), and CXCL7 (log_2_ FC = −4.674, *p* < 0.001) being the most significantly downregulated. The top 10 upregulated and top 10 downregulated proteins are listed in **Table 2**, and volcano plot of the differential proteins is shown in **Supplementary Figure 2**.

**Table 2:** Statistics of differential proteins between total patients with GD and healthy controls.

Protein name	Accession number	log_2_ FC	*p*-Value	*Q* value	Class
Microtubule-associated protein 1S (MAP1S)	Q66K74	4.669	0.009	0.004	Up
Vesicle-associated membrane proteins 8 (VAMP8)	Q9BV40	3.961	0.001	0.002	Up
Eukaryotic translation initiation factor 5 (IF5)	P55010	2.590	0.024	0.033	Up
Pulmonary surfactant-associated protein B (PSPB)	P07988	2.469	0.009	0.030	Up
Fibrinogen-like protein 1 (FGL1)	Q08830	1.908	0.014	0.040	Up
Acid ceramidase (ASAH1)	Q13510	1.664	0.007	0.023	Up
Peroxidasin homolog (PXDN)	Q92626	1.648	0.016	0.035	Up
Sex hormone-binding globulin (SHBG)	P04278	1.595	0.038	0.048	Up
ATP-binding cassette sub-family F member 2 (ABCF2)	Q9UG63	1.247	0.011	0.036	Up
Insulin-like growth factor-binding protein 2 (IBP2)	P18065	1.019	0.035	0.043	Up
Ras suppressor protein 1 (RSU1)	Q15404	−6.797	<0.001	0.010	Down
Actin, cytoplasmic 1 (ACTB)	P60709	−4.795	<0.001	0.010	Down
Platelet basic protein (CXCL7)	P02775	−4.674	<0.001	0.010	Down
l-Lactate dehydrogenase A chain (LDHA)	P00338	−2.904	<0.001	0.010	Down
Dopamine beta-hydroxylase (DOPO)	P09172	−2.151	<0.001	0.005	Down
Desmocollin-3 (DSC3)	Q14574	−1.846	<0.001	0.008	Down
Clusterin (CLUS)	P10909	−1.211	<0.001	0.001	Down
Kallistatin (KAIN)	P29622	−1.117	<0.001	<0.001	Down
Cartilage acidic protein 1 (CRAC1)	Q9NQ79	−1.084	<0.001	0.010	Down
C4b-binding protein beta chain (C4BPB)	P20851	−1.065	<0.001	0.010	Down

Q value means that the p-value is adjusted for the false discovery rate (FDR). All proteins in the table were screened based on both p-value and Q value <0.5. Only the 10 most upregulated and 10 most downregulated differential proteins are listed.

GD, Graves’ disease.

**Figure 1:**
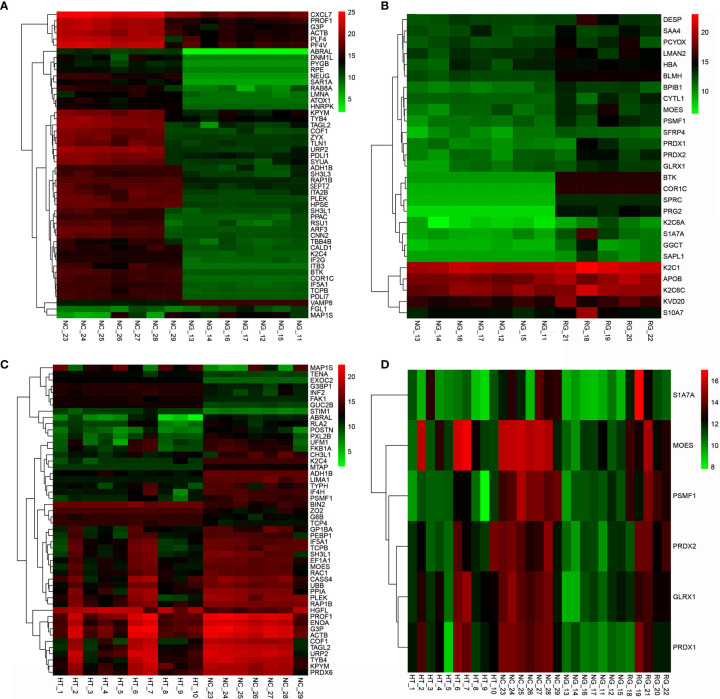
Clustering heat map of differential proteins between AITD and NC groups **(A)**, NG group *vs.* NC group **(B)**, NG group *vs.* RG group **(C)**, and HT group *vs.* NC group **(D)**. NG group *vs.* RG group *vs.* HT group *vs.* NC group. AITD, autoimmune thyroid disease; NC, normal control; NG, newly diagnosed Grave’s disease; RG, refractory Grave’s disease; HT, Hashimoto’s thyroiditis.

The subgroup analysis of the GD patients showed that the NG group had more differential proteins than the RG group compared with the NC group. Specifically, screening all differential proteins with *Q* value (adjusted *p*-value) <0.5 indicates that 127 proteins were downregulated, and VAMP8 (log_2_ FC = 3.216, *p* = 0.003, *Q* = 0.02) was the most upregulated in the NG group (**Figure 1A**). Euclidean distance and hierarchical clustering of differential proteins showed almost no significant difference between the RG group and NC group except that only PTN12 (log_2_ FC = −2.154, *p* < 0.001, *Q* = 0.006) was significantly downregulated. The analysis of differential proteins between the NG and RG groups found that the levels of CORO1C (log_2_ FC = 7.185, *p* = 0.003, *Q* = 0.006), BTK (log_2_ FC = 6.394, *p* = 0.017, *Q* = 0.034), and PRG2 (log_2_ FC = 6.245, *p* = 0.016, *Q* = 0.012) were significantly higher in the RG group than in the NG group (**Figure 1B**).

Further analysis of differential proteins showed that in the HT group, seven proteins including HGFL (log_2_ FC = 2.766, *p* = 0.001, *Q* = 0.036), FAK1 (log_2_ FC = 2.213, *p* < 0.001, *Q* = 0.015), FBN1 (log_2_ FC = 1.745, *p* < 0.001, *Q* = 0.017), PTN12 (log_2_ FC = 1.624, *p* < 0.001, *Q* = 0.021), C1QB (log_2_ FC = 1.048, *p* < 0.001, *Q* = 0.015), and C1QC (log_2_ FC = 1.029, *p* < 0.001, *Q* = 0.008) were upregulated. Moreover, nine proteins such as PSMF1 (log_2_ FC = −3.591, *p* < 0.001, *Q* = 0.012), PXL2B (log_2_ FC = −2.622, *p* = 0.001, *Q* = 0.037), CYTM (log_2_ FC = −1.609, *p* < 0.001, *Q* = 0.021), and TRFE (log_2_ FC = −1.009, *p* = 0.001, *Q* = 0.016) were significantly downregulated (**Table 3** and **Figure 1C**). Differential proteins between all AITDs patients and NC were shown in cluster map (**Figure 1D**). The NG group and the NC group showed the most significant difference, while the RG group and the NC group showed the least significant difference.

**Table 3:** Statistics of differential proteins between patients with HT and healthy controls.

Protein name	Accession number	log_2_ FC	*p*-Value	*Q* value	Class
Hepatocyte growth factor-like protein (HGFL)	P26927	2.766	0.001	0.036	Up
Focal adhesion kinase 1 (FAK1)	Q05397	2.213	<0.001	0.015	Up
Fibrillin-1 (FBN1)	P35555	1.745	<0.001	0.017	Up
Tyrosine-protein phosphatase non-receptor type 12 (PTN12)	Q05209	1.624	<0.001	0.021	Up
Acidic fibroblast growth factor intracellular-binding protein (FIBP)	O43427	1.408	<0.001	0.023	Up
Complement C1q subcomponent subunit B (C1QB)	P02746	1.048	<0.001	0.015	Up
Complement C1q subcomponent subunit C (C1QC)	P02747	1.029	<0.001	0.008	Up
Proteasome inhibitor PI31 subunit (PSMF1)	Q92530	−3.591	<0.001	0.012	Down
Prostamide/prostaglandin F synthase (PXL2B)	Q8TBF2	−2.622	0.001	0.037	Down
Cystatin-M (CYTM)	Q15828	−1.609	<0.001	0.021	Down
Serotransferrin (TRFE)	P02787	−1.009	<0.001	0.016	Down
Alpha-2-HS-glycoprotein (FETUA)	P02765	−1.088	<0.001	0.021	Down
Extracellular matrix protein 1 (ECM1)	Q16610	−1.129	<0.001	0.021	Down
Albumin (ALBU)	P02768	−1.015	<0.001	0.023	Down
Beta-2-glycoprotein 1 (APOH)	P02749	−1.047	<0.001	0.030	Down
Serum paraoxonase/arylesterase 1 (PON1)	P27169	−1.240	0.001	0.032	Down

Q value means that the p-value is adjusted for the false discovery rate (FDR). All proteins in the table were screened based on both p-value and Q value <0.5.

HT, Hashimoto’s thyroiditis.

### Function Analysis of Differential Proteins

**Figure 2:**
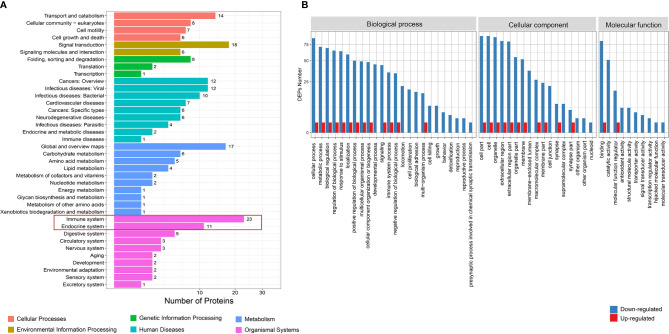
Function analysis of differential proteins between total GD and NC groups. **(A)** KEGG enrichment analysis. **(B)** GO analysis for upregulated and downregulated proteins. GD, Graves’ disease; NC, normal control; KEGG, Kyoto Encyclopedia of Genes and Genomes; GO, Gene Ontology.

**Figure 3:**
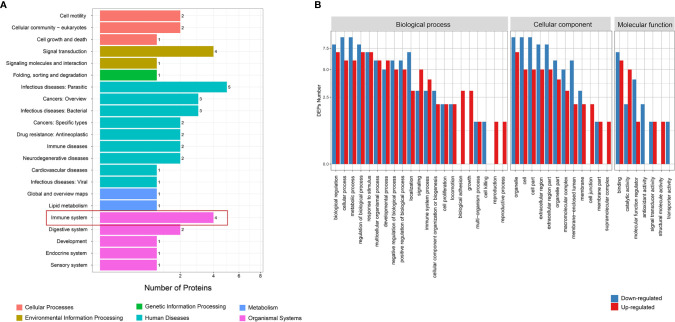
Function analysis of differential proteins between HT and NC groups. **(A)** KEGG enrichment analysis. **(B)** GO analysis for upregulated and downregulated proteins. HT, Hashimoto’s thyroiditis; NC, normal control; KEGG, Kyoto Encyclopedia of Genes and Genomes; GO, Gene Ontology.

KEGG analysis found that the differential proteins were mainly concentrated in the immune system and endocrine system, which is consistent with the etiological background of AITDs (**Figure 2A**). GO analysis found that the differential proteins between the GD and NC groups mainly manifested in cellular process, metabolic process, and biological regulation (**Figure 2B**). KOG analysis found that the differential proteins between the GD and NC groups were mainly enriched in posttranslational modification, protein turnover, and chaperones (**Supplementary Figure 3A**). Further pathway enrichment analysis revealed that most of the differential proteins were enriched in the metabolic pathways and glycolysis/gluconeogenesis pathways (**Supplementary Figure 3B**). Similarly, KEGG analysis of the differential proteins between the HT and NC groups also found that they were mainly enriched in the immune system (**Figure 3A**). GO analysis results showed that these differential proteins were mainly concentrated in biological regulation, metabolic process, biological process regulation, and immune system among other pathways (**Figure 3B**). KOG analysis found that these proteins were mainly enriched in the signal transduction mechanisms (**Supplementary Figure 4A**), and further KEGG analysis revealed that the exosome proteins in HT patients were related to other autoimmune diseases such as systemic lupus erythematosus (SLE) (**Supplementary Figure 4B**). Comprehensively, the functional analysis suggested that the exosomal differential proteins in patients with AITDs were mainly immune-related.

## Discussion

A typical exosome is a small membrane transporter with a diameter of 50–150 nm and a “cup-shaped” bilayer lipid membrane structure under transmission electron microscopy. In the body, exosomes are widely derived from a variety of cells in the body, such as macrophages, endothelial cells, liver cells, nerve cells, fat cells, and immune cells (19). Exosomes are actively secreted by these cells and continuously exist in the circulation or enter the extracellular microenvironment (20). Importantly, exosomes are shown to carry biologically active molecules such as proteins, mRNA, miRNA, and lipid from donor cells and therefore play important roles in active substances and information exchanges between cells (21, 22). Under different stimuli or certain physiological and pathological conditions, the composition and quantity of exosomes will change significantly (23, 24). Exosomes contain certain specific protein components that have been proven to be closely related to the etiology, development, and outcome of diseases (25–27). The etiology of AITDs is very complex and involves multiple factors such as genetics, environment, gender, and immunity (28, 29). Although AITDs are organ-specific autoimmune diseases, they are prone to cause systemic immune imbalance and tend to co-occur with other autoimmune diseases (30). Therefore, we aimed to investigate whether circulating exosomes are responsible for important information communication in AITD patients. Our experiments for the first time showed a significant difference in the exosomal protein profiles between patients with AITDs (both GD and HT) and healthy controls and revealed that these differential proteins were mainly concentrated in the immune system and metabolic pathways. These results indicated that plasma exosomes may be a bridge between organ-specific autoimmune thyroiditis and systemic immune imbalance, providing new perspectives and ideas for further exploration of the pathologies of GD and HT.

Many differential proteins between the GD group and the NC group were found by DIA (SWATH-MS) method, which is an emerging and high-efficient method for protein profiling. Among them, the most significantly upregulated proteins were MAP1S, VAMP8, and IF5; and the most significantly downregulated proteins were RSU1, ACTB, and CXCL7. These proteins were mainly enriched in the immune system, suggesting that the exosomal proteins may participate in the systemic immune imbalance and thus promote the development of the disease. VAMP8 promotes the secretion of TNF-α, facilitates T lymphocyte cytotoxicity (31), and engages in immune cell extravasation during inflammatory response (32). Although T lymphocyte infiltration of the thyroid is a well-established pathological feature of AITDs, VAMP8-mediated GD is reported for the first time in this study. MAP1S is an autophagy-related protein that mediates mitochondrial aggregation genome destruction and plays a role in apoptosis (33). The level of autophagy in GD is reported to have significantly increased (34), and our study revealed for the first time that high levels of MAP1S in circulating exosomes may be one of the stimulating factors for high autophagy levels in the GD patients. CXCL7, an important chemoattractant cytokine, was found to be downregulated in exosomal proteins from patients with GD in our study. However, Van et al. analyzed the genome of adult with AITDs and found that *CXCL7* gene was overexpressed in monocytes (35). The role of *CXCL7* gene in the pathological mechanism of AITD may be very complicated and requires deeper research. There were also many differential proteins between the HT group and NC group, although their number was much smaller than that between the GD group and NC group. Among them, HGFL, FAK1, FBN1, PTN12, C1QC, and C1QB were significantly higher in exosomes of HT patients, while PSMF1, PXL2B, CYTM, TRFE, and FETUA were significantly lower. These proteins are also enriched in the immune system and metabolic system and are closely related to cancer and other autoimmune diseases like SLE. FBN1 regulates the bioavailability and storage of TGF-β superfamily growth factors (36), which may therefore mediate the development of HT.

In conclusion, our study provides some new perspectives for deepening our understanding of AITDs. Plasma exosomal proteins may play an important role in the systemic immune imbalance of patients with AITDs. Nevertheless, the present study has certain shortcomings. Although an advanced protein profiling method was used, the sample size in this study still needs to be further expanded. Moreover, the specific role of these differential proteins needs to be investigated in more depth in future.

## Data Availability Statement

The original contributions presented in the study are included in the article/**Supplementary Material**. Further inquiries can be directed to the corresponding author.

## Ethics Statement

The studies involving human participants were reviewed and approved by The Ethics Committee of Zhoupu Hospital. The patients/participants provided their written informed consent to participate in this study.

## Author Contributions

XJ was responsible for the design, operation, and data analysis of the experiment. TZ was responsible for the sample collection, and J-aZ was responsible for the overall direction of the research. All authors contributed to the article and approved the submitted version.

## Funding

The present work was supported by grants from the National Natural Science Foundation of China (No. 81873636 and No. 81900710), Pudong New Area Health Commission key sub-specialty (PWZy2020-12), Shanghai University of Medicine & Health Sciences hundreds of Talented Teachers Project (No. ZPBRK-20-03), Clinical Research Center of thyroid diseases of Shanghai Health Medical College (20MC20200002), and Project of Shanghai Medical Key Specialty (No. ZK2019C09).

## Conflict of Interest

The authors declare that the research was conducted in the absence of any commercial or financial relationships that could be construed as a potential conflict of interest.

## Publisher’s Note

All claims expressed in this article are solely those of the authors and do not necessarily represent those of their affiliated organizations, or those of the publisher, the editors and the reviewers. Any product that may be evaluated in this article, or claim that may be made by its manufacturer, is not guaranteed or endorsed by the publisher.
