# Supplementary material for: Circulating Exosome Involves in the Pathogenesis of Autoimmune Thyroid Diseases Through Immunomodulatory Proteins
Source: Front Immunol. 2021 Nov 11;12:730089. doi: 10.3389/fimmu.2021.730089 (PMC8636008; doi:10.3389/fimmu.2021.730089)
Supplement: Supplementary file 1 [file DataSheet_1.pdf]

Supplementary material

Table S1. The number of peptides and proteins identified in each sample

Samples	No. of peptides	No. of proteins
HT_1	3736	516
HT_2	5057	840
HT_3	3619	499
HT_4	4144	623
HT_5	3481	447
HT_6	5509	963
HT_7	5902	1016
HT_8	4052	551
HT_9	4024	589
HT_10	4126	634
GD_11	4085	583
GD_12	4134	593
GD_13	3488	456
GD_14	3379	446
GD_15	4926	807
GD_16	3978	555
GD_17	3697	499
GD_18	3693	495
GD_19	3915	538
GD_20	4173	595
GD_21	4079	590
GD_22	4111	600
NC_23	5728	986
NC_24	5797	992
NC_25	5360	897
NC_26	4735	771
NC_27	5589	948
NC_28	5621	947
NC_29	3950	555

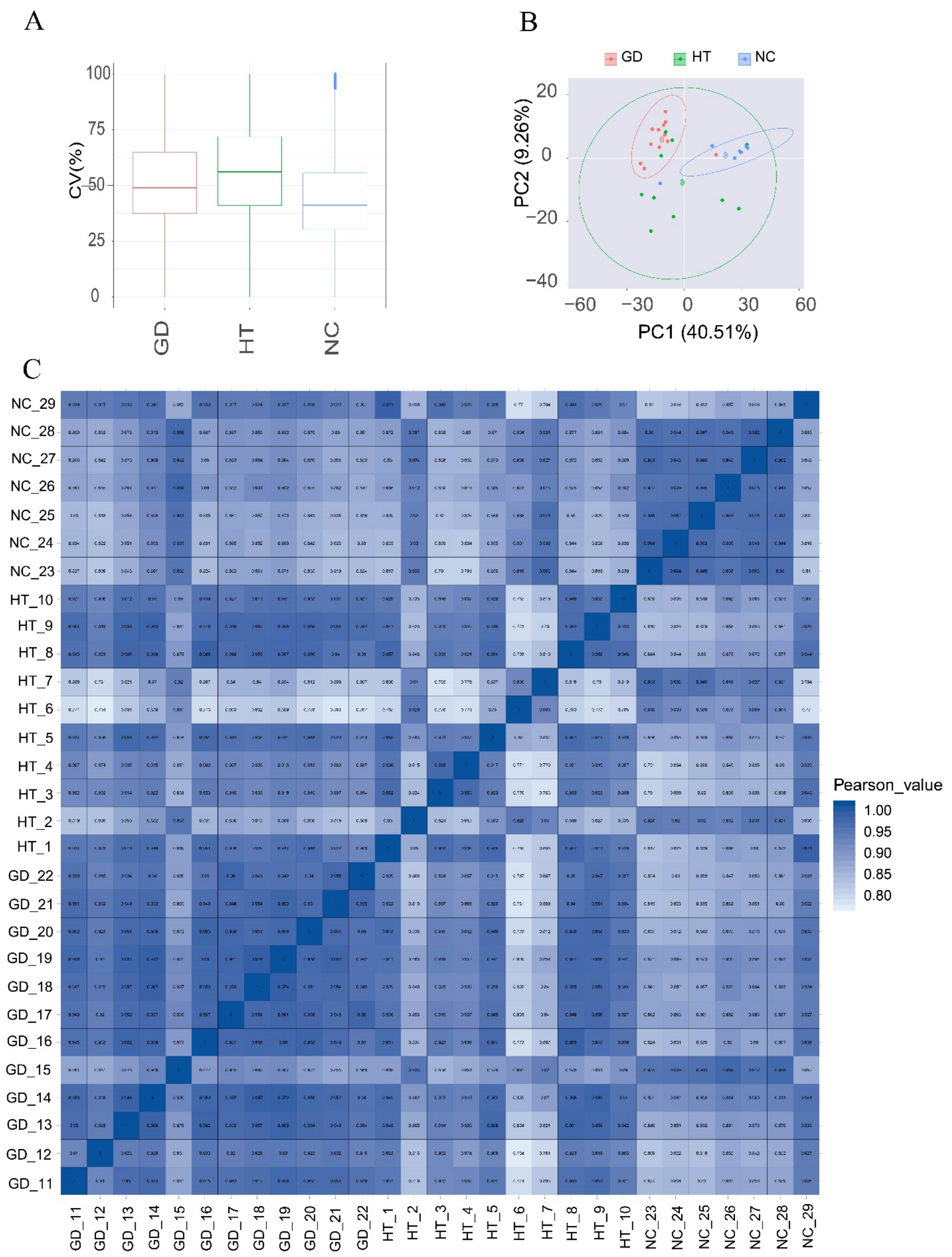

Fig. S1 Protein consistency detection and quality control. (A) Intra-group coefficient of variation (CV) of different sample groups. The X-axis denotes the sample group and the Y-axis denotes the corresponding CV; (B) Principal Component Analysis (PCA) of different sample groups; (C) Quantitative correlation of samples identified using Pearson correlation coefficient.

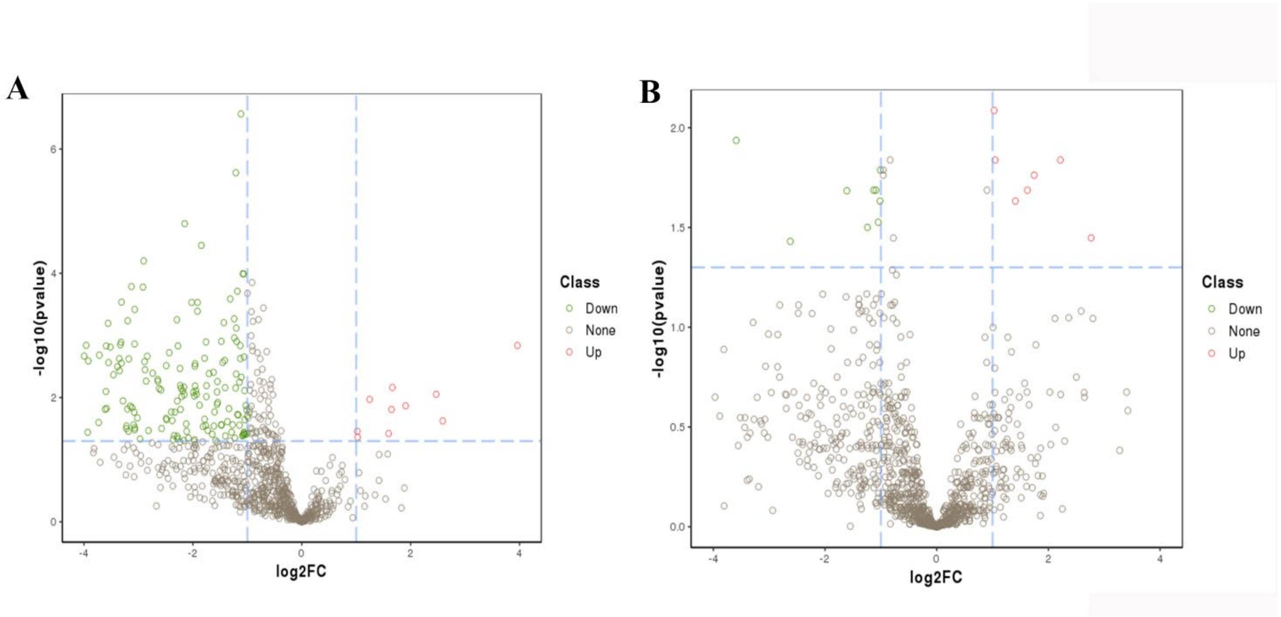

Fig. S2 Volcano map of differential proteins. (A) GD *vs.* NC groups; (B) HT *vs.* NC groups. The X-axis of the graph is the protein fold change (log2), and the Y-axis is the corresponding -log10 (*P* value). The red dots indicate significantly up-regulated proteins, the green dots indicate significantly down-regulated proteins, and the grey dots indicate proteins without significant change.

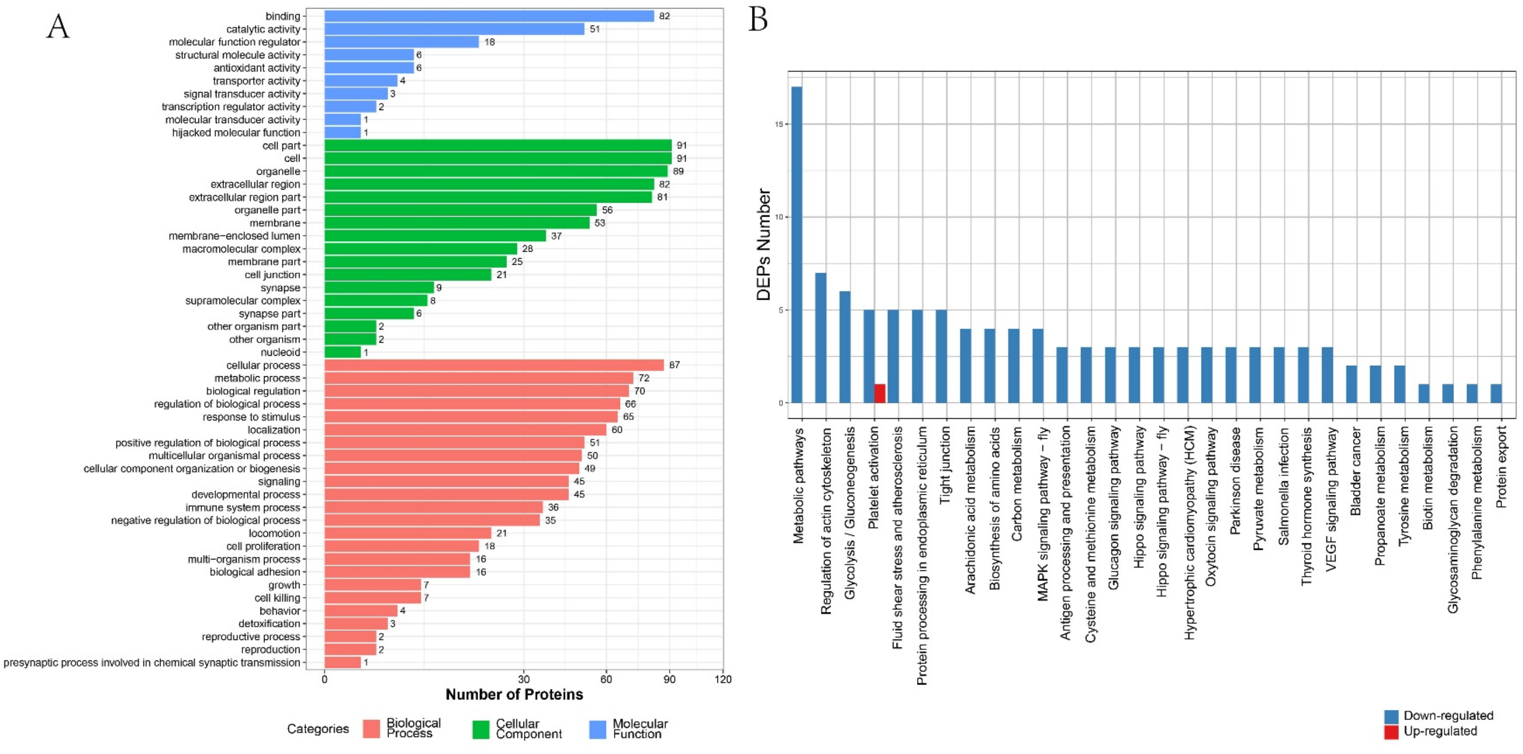

Fig. S3 Function analysis of differential proteins between total GD and NC groups. (A) KOG enrichment analysis; (B) KEGG analysis for up-regulated and down-regulated proteins;

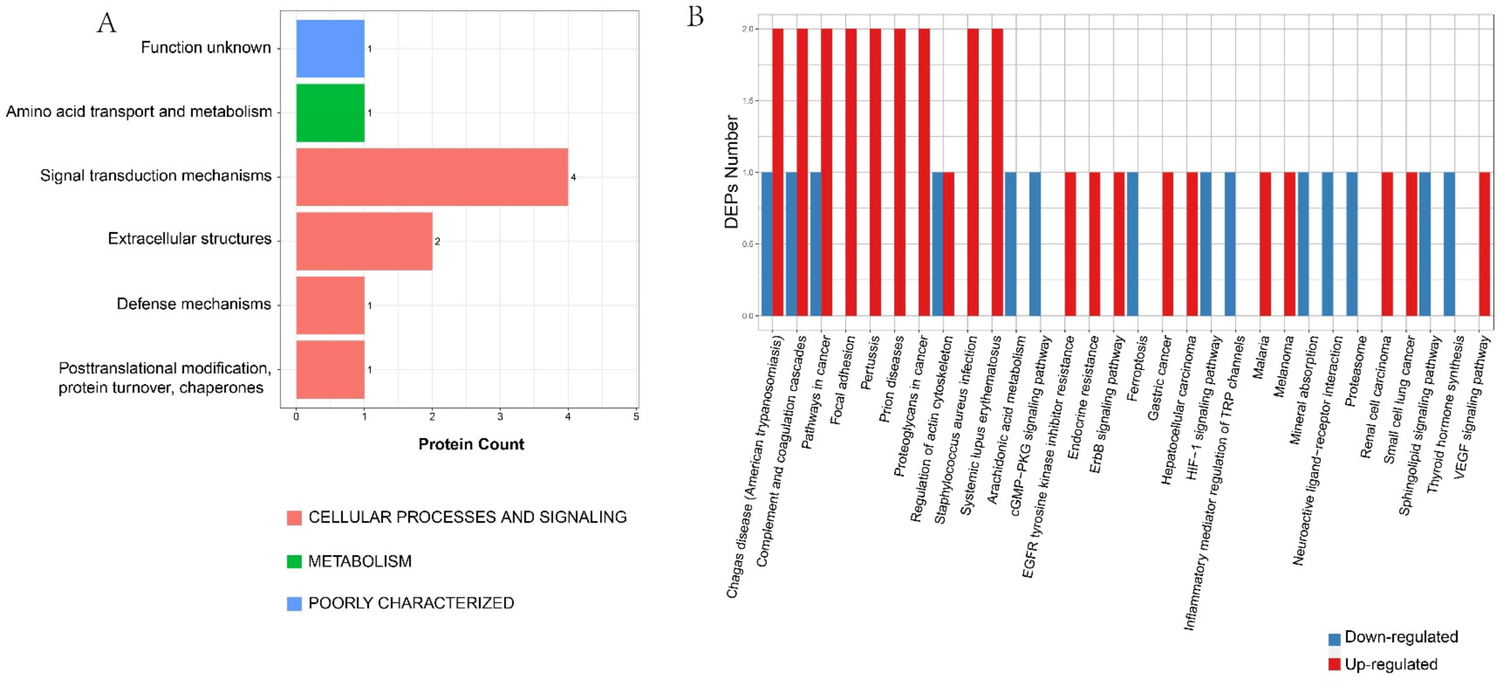

Fig. S4 Function analysis of differential proteins between total HT and NC groups. (A) KOG enrichment analysis; (B) KEGG analysis for up-regulated and down-regulated proteins;
